# Supplementary material for: Modelling Skylarks (Alauda arvensis) to Predict Impacts of Changes in Land Management and Policy: Development and Testing of an Agent-Based Model
Source: PLoS One. 2013 Jun 6;8(6):e65803. doi: 10.1371/journal.pone.0065803 (PMC3675089; doi:10.1371/journal.pone.0065803)
Supplement: Supporting Information S4 — The skylark ODdox as a zipped archive. (ZIP) [file pone.0065803.s004.zip › Skylark_ODdox/class_skylark___population___manager-members.html]

ALMaSS Skylark ODdox: Member List


ALMaSS Skylark ODdox
 2.0


- Main Page
- Related Pages
- Classes
- Files

- Class List
- Class Index
- Class Hierarchy
- Class Members

Skylark\_Population\_Manager Member List

This is the complete list of members for Skylark\_Population\_Manager, including all inherited members.

|  |  |  |
| --- | --- | --- |
| AddStriglingMort(int lifestage) | Skylark\_Population\_Manager | inline |
| AlFreq | Population\_Manager |  |
| BeforeStepActions | Population\_Manager | protected |
| BeginningOfMonth() | Population\_Manager |  |
| BreedingPairsOutput(int Time) | Skylark\_Population\_Manager | virtual |
| BreedingSuccessProbeOutput(double, int, int, int, int, int, int, int) | Skylark\_Population\_Manager | virtual |
| Catastrophe() | Skylark\_Population\_Manager | protectedvirtual |
| Population\_Manager::Catastrophe(int) | Population\_Manager | inlinevirtual |
| CheckXY(int l, int i) | Population\_Manager |  |
| CIPEGridOutputPrb | Population\_Manager | protected |
| CIPEGridOutputPrbB | Population\_Manager | protected |
| CloseTheCIPEGridOutputProbe() | Population\_Manager |  |
| CloseTheMonthlyRipleysOutputProbe() | Population\_Manager |  |
| CloseTheReallyBigOutputProbe() | Population\_Manager |  |
| CloseTheRipleysOutputProbe() | Population\_Manager |  |
| Counts | Population\_Manager |  |
| CreateObjects(int ob\_type, TAnimal \*pTAo, void \*null, Skylark\_struct \*data, int number) | Skylark\_Population\_Manager |  |
| DisplayLocations() | Population\_Manager | virtual |
| DoAfter() | Population\_Manager | protectedvirtual |
| DoAlmostLast() | Population\_Manager | protectedvirtual |
| DoBefore() | Population\_Manager | protectedvirtual |
| DoFirst() | Skylark\_Population\_Manager | protectedvirtual |
| DoLast() | Population\_Manager | protectedvirtual |
| EMi | Skylark\_Population\_Manager | protected |
| F\_Mig\_Mort | Skylark\_Population\_Manager | protected |
| FindClosest(int x, int y, unsigned Type) | Population\_Manager |  |
| FledgelingProbeOutput(int Total, int Time) | Skylark\_Population\_Manager | virtual |
| GeneticsResultsOutput(FILE \*, unsigned) | Population\_Manager | inlinevirtual |
| ImpactedProbe() | Population\_Manager | virtual |
| ImpactProbeReport(int a\_Time) | Population\_Manager |  |
| incNoChickDeaths() | Skylark\_Population\_Manager | inline |
| incNoFledgeDeaths() | Skylark\_Population\_Manager | inline |
| incNoPestEffects() | Skylark\_Population\_Manager | inline |
| incTotalEggs(int eggs) | Skylark\_Population\_Manager | inline |
| incTotalNestlings() | Skylark\_Population\_Manager | inline |
| incTotalPrefledgelings() | Skylark\_Population\_Manager | inline |
| IndexArrayX | Population\_Manager |  |
| Init(void) | Skylark\_Population\_Manager | virtual |
| IsBadWeather() | Skylark\_Population\_Manager | inline |
| IsLast(unsigned listindex) | Population\_Manager | inline |
| LamdaBirth(int x, int y) | Population\_Manager | inline |
| LamdaBirth(int x, int y, int z) | Population\_Manager | inline |
| LamdaClear() | Population\_Manager | inline |
| LamdaDeath(int x, int y) | Population\_Manager | inline |
| LamdaDumpOutput() | Population\_Manager |  |
| lamdagrid | Population\_Manager | protected |
| LoadParameters() | Skylark\_Population\_Manager | protected |
| LOG(const char \*fname) | Population\_Manager |  |
| m\_AlleleFreqsFile | Population\_Manager | protected |
| m\_catastrophestartyear | Population\_Manager | protected |
| m\_cgridcount | Population\_Manager | protected |
| m\_cgridcountwidth | Population\_Manager | protected |
| m\_cipegridsize | Population\_Manager | protected |
| m\_EasyPopRes | Population\_Manager | protected |
| m\_GeneticsFile | Population\_Manager | protected |
| m\_gridcount | Population\_Manager | protected |
| m\_gridcountsize | Population\_Manager | protected |
| m\_gridcountwidth | Population\_Manager | protected |
| m\_IsBadWeather | Skylark\_Population\_Manager | protected |
| m\_ListNameLength | Population\_Manager | protected |
| m\_ListNames | Population\_Manager | protected |
| m\_MainForm | Population\_Manager |  |
| M\_Mig\_Mort | Skylark\_Population\_Manager | protected |
| m\_NoChickDeaths | Skylark\_Population\_Manager | protected |
| m\_NoFledgeDeaths | Skylark\_Population\_Manager | protected |
| m\_NoPestEffects | Skylark\_Population\_Manager | protected |
| m\_NoProbes | Population\_Manager | protected |
| m\_SimulationName | Population\_Manager |  |
| m\_StepSize | Population\_Manager | protected |
| m\_StriglingMort | Skylark\_Population\_Manager | protected |
| m\_TheLandscape | Population\_Manager |  |
| m\_TotalEggs | Skylark\_Population\_Manager | protected |
| m\_TotalNestlings | Skylark\_Population\_Manager | protected |
| m\_TotalPrefledgelings | Skylark\_Population\_Manager | protected |
| NWordOutput() | Population\_Manager | protected |
| NWordOutputPrb | Population\_Manager | protected |
| OpenTheBreedingPairsProbe() | Skylark\_Population\_Manager | virtual |
| OpenTheBreedingSuccessProbe() | Skylark\_Population\_Manager | virtual |
| OpenTheCIPEGridOutputProbe() | Population\_Manager |  |
| OpenTheFledgelingProbe() | Skylark\_Population\_Manager | virtual |
| OpenTheMonthlyRipleysOutputProbe() | Population\_Manager |  |
| OpenTheReallyBigProbe() | Population\_Manager |  |
| OpenTheRipleysOutputProbe() | Population\_Manager |  |
| Population\_Manager(Landscape \*L) | Population\_Manager |  |
| Probe(int ListIndex, probe\_data \*p\_TheProbe) | Population\_Manager | virtual |
| ProbeFileInput(char \*p\_Filename, int p\_ProbeNo) | Population\_Manager |  |
| ProbePOM(int ListIndex, probe\_data \*p\_TheProbe) | Skylark\_Population\_Manager | protected |
| ProbeReport(int a\_time) | Population\_Manager |  |
| ProbeReportPOM(int a\_time) | Skylark\_Population\_Manager | protected |
| ProbeReportTimed(int a\_time) | Population\_Manager |  |
| ProbesSet | Population\_Manager |  |
| ReallyBigOutputPrb | Population\_Manager | protected |
| ReHouse() | Skylark\_Population\_Manager | protectedvirtual |
| RipleysOutputPrb | Population\_Manager | protected |
| RipleysOutputPrb1 | Population\_Manager | protected |
| RipleysOutputPrb10 | Population\_Manager | protected |
| RipleysOutputPrb11 | Population\_Manager | protected |
| RipleysOutputPrb12 | Population\_Manager | protected |
| RipleysOutputPrb2 | Population\_Manager | protected |
| RipleysOutputPrb3 | Population\_Manager | protected |
| RipleysOutputPrb4 | Population\_Manager | protected |
| RipleysOutputPrb5 | Population\_Manager | protected |
| RipleysOutputPrb6 | Population\_Manager | protected |
| RipleysOutputPrb7 | Population\_Manager | protected |
| RipleysOutputPrb8 | Population\_Manager | protected |
| RipleysOutputPrb9 | Population\_Manager | protected |
| Run(int NoTSteps) | Population\_Manager | virtual |
| SetF\_Mig\_Mort(int m) | Skylark\_Population\_Manager | inline |
| SetM\_Mig\_Mort(int m) | Skylark\_Population\_Manager | inline |
| SetNoProbes(int a\_pn) | Population\_Manager | inline |
| Shuffle(unsigned Type) | Population\_Manager | protected |
| Shuffle\_or\_Sort(unsigned Type) | Population\_Manager | protected |
| SimH | Population\_Manager |  |
| SimHH | Population\_Manager |  |
| SimW | Population\_Manager |  |
| SimWH | Population\_Manager |  |
| SKPOM1 | Skylark\_Population\_Manager | protected |
| SKPOM2 | Skylark\_Population\_Manager | protected |
| Skylark\_Population\_Manager(Landscape \*L) | Skylark\_Population\_Manager |  |
| SortState(unsigned Type) | Population\_Manager | protected |
| SortStateR(unsigned Type) | Population\_Manager | protected |
| SortX(unsigned Type) | Population\_Manager | protected |
| SortXIndex(unsigned Type) | Population\_Manager | protected |
| SortY(unsigned Type) | Population\_Manager | protected |
| SpeciesSpecificReporting(int a\_species, int a\_time) | Population\_Manager |  |
| StateList | Population\_Manager | protected |
| StateNames | Population\_Manager | protected |
| StateNamesLength | Population\_Manager | protected |
| StepFinished() | Population\_Manager | protectedvirtual |
| SupplyCovPosx(int) | Population\_Manager | inlinevirtual |
| SupplyCovPosy(int) | Population\_Manager | inlinevirtual |
| SupplyEMi() | Skylark\_Population\_Manager | inline |
| SupplyF\_Mig\_Mort() | Skylark\_Population\_Manager | inline |
| SupplyListIndexSize() | Population\_Manager | inline |
| SupplyListName(int i) | Population\_Manager | inline |
| SupplyListNameLength() | Population\_Manager | inline |
| SupplyListSize(unsigned listindex) | Population\_Manager | inline |
| SupplyLocXY(unsigned listindex, unsigned j, int &x, int &y) | Population\_Manager | inlinevirtual |
| SupplyM\_Mig\_Mort() | Skylark\_Population\_Manager | inline |
| SupplyNoTerritories() | Skylark\_Population\_Manager |  |
| SupplyPegPosx(int) | Population\_Manager | inlinevirtual |
| SupplyPegPosy(int) | Population\_Manager | inlinevirtual |
| SupplySimH() | Population\_Manager | inline |
| SupplySimW() | Population\_Manager | inline |
| SupplyState(unsigned listindex, unsigned j) | Population\_Manager | inline |
| SupplyStateList() | Population\_Manager | inline |
| SupplyStateNames(int i) | Population\_Manager | inline |
| SupplyStateNamesLength() | Population\_Manager | inline |
| SupplyStepSize() | Population\_Manager | inline |
| TestFile | Population\_Manager | protected |
| TestFile2 | Population\_Manager | protected |
| TheArray | Population\_Manager | protected |
| TheBreedingFemalesProbe(int ProbeNo) | Skylark\_Population\_Manager | virtual |
| TheBreedingSuccessProbe(int &BreedingFemales, int &YoungOfTheYear, int &TotalPop, int &TotalFemales, int &TotalMales, int &BreedingAttempts) | Skylark\_Population\_Manager | virtual |
| TheCIPEGridOutputProbe() | Skylark\_Population\_Manager | virtual |
| TheFledgelingProbe() | Skylark\_Population\_Manager | virtual |
| TheGeneticProbe(unsigned, int, unsigned &) | Population\_Manager | inlinevirtual |
| TheNWordOutputProbe() | Skylark\_Population\_Manager | virtual |
| TheProbe | Population\_Manager |  |
| TheReallyBigOutputProbe() | Population\_Manager | virtual |
| TheRipleysOutputProbe(FILE \*a\_prb) | Skylark\_Population\_Manager | virtual |
| TheSkylarkTerrs | Skylark\_Population\_Manager |  |
| TheSkylarkTerrsSupply\_quality(int) | Skylark\_Population\_Manager |  |
| TheSkylarkTerrsSupply\_size(int) | Skylark\_Population\_Manager |  |
| TheSkylarkTerrsSupply\_x(int) | Skylark\_Population\_Manager |  |
| TheSkylarkTerrsSupply\_y(int) | Skylark\_Population\_Manager |  |
| VegTypeFledgelings | Skylark\_Population\_Manager |  |
| WriteSKPOM1(int n, int n2) | Skylark\_Population\_Manager | inline |
| WriteSKPOM2(int n, int n2) | Skylark\_Population\_Manager | inline |
| ~Population\_Manager(void) | Population\_Manager | virtual |
| ~Skylark\_Population\_Manager(void) | Skylark\_Population\_Manager | virtual |


- Generated on Thu Jan 10 2013 13:15:38 for ALMaSS Skylark ODdox by
   1.8.1.1
